# A portable NMR platform with arbitrary phase control and temperature compensation

**DOI:** 10.5194/mr-3-77-2022

**Published:** 2022-05-16

**Authors:** Qing Yang, Jianyu Zhao, Frederik Dreyer, Daniel Krüger, Jens Anders

**Affiliations:** 1 Institute of Smart Sensors, University of Stuttgart, Pfaffenwaldring 47, 70569 Stuttgart, Germany; 2 John A. Paulson School of Engineering and Applied Sciences, Harvard University, Cambridge, MA 02138, United States; 3 Center for Integrated Quantum Science and Technology (IQ^ST^), Stuttgart, Germany​​​​​​​

## Abstract

In this paper, we present a custom-designed nuclear magnetic resonance (NMR) platform based on a broadband complementary metal–oxide–semiconductor (CMOS) NMR-on-a-chip transceiver and a synchronous reference signal generator, which features arbitrary phase control of the excitation pulse in combination with phase-coherent detection at a non-zero intermediate frequency (IF). Moreover, the presented direct digital synthesis (DDS)-based frequency generator enables a digital temperature compensation scheme similar to classical field locking without the need for additional hardware. NMR spectroscopy and relaxometry measurements verify the functionality of the proposed frequency reference and temperature compensation scheme as well as the overall state-of-the-art performance of the presented system.

## 1 Introduction

Nuclear magnetic resonance (NMR) is one of the most powerful analytical methods that allows for the direct measurement of molecular information. Due to its non-invasive nature and the possibility of measuring the NMR signal contactless, NMR is widely used in biomedicine (Peng et al., 2014; Chen et al., 2021), chemistry (Singh and Blumich, 2018), agriculture (Colnago et al., 2021), and industrial applications (Rudszuck et al., 2021). Over the last 10 to 15 years, with technology advancements in the fields of magnet design, pulse sequences, and electronics, NMR has seen two major areas of evolution. In high-field NMR, the increasing requirements for sensitivity and resolution lead to sophisticated and cumbersome NMR devices based on superconducting magnets with higher and higher magnetic field strength and very high field homogeneity (Gan et al., 2017). These devices are capable of chemical structure analysis and medical imaging with unprecedented spectral and spatial resolution. In low-field NMR, the development of chip-integrated CMOS-based NMR transceiver electronics (NMR-on-a-chip) (Sun et al., 2009; Ha et al., 2014; Grisi et al., 2015; Lei et al., 2016a; Handwerker et al., 2016; Bürkle et al., 2020) has led to portable NMR (pNMR) detection platforms based on permanent magnets, which are suitable for point-of-care applications (Lee et al., 2008; Liong et al., 2013; Ha et al., 2014; Lei et al., 2015, 2017, 2020).

As detailed in two recent review articles (Anders and Lips, 2019; Anders et al., 2021), the NMR-on-a-chip approach allows for integrating the entire NMR console on a tiny footprint of a few square millimeters. To the best of our knowledge, the original idea of designing CMOS-based NMR electronics was presented by Boero et al. (1998). Since then, the idea of integrating planar on-chip microcoils with CMOS transceivers has been widely employed (Lei et al., 2016b; Grisi et al., 2017; Handwerker et al., 2020) to produce miniaturized NMR detectors with very small detection volumes and very good spin sensitivities. Initially, the NMR-on-a-chip detectors were designed for operation inside conventional superconducting NMR magnets, focusing on the miniaturization and parallelization of the NMR receiver (RX) (Boero et al., 2001; Anders and Boero, 2008; Kim et al., 2010; Anders et al., 2011). Later, with the advent of small-sized permanent NMR magnets, fully-integrated NMR transceiver – i.e., combined transmitter and receiver – chips were developed to realize portable, low-field NMR detection platforms (Liu et al., 2008; Sun et al., 2011; Ha et al., 2014). The transceivers were then also extended to high-field NMR by the use of on-chip wide-range frequency synthesizers (Kim et al., 2012; Grisi et al., 2015; Handwerker et al., 2016). More recent developments include improvements in the driving strength of the NMR-on-a-chip transceivers by the use of high-voltage CMOS technologies (Bürkle et al., 2020, 2021) and the co-integration of electron spin resonance (EPR) electronics on a single chip to perform on-chip dynamic nuclear polarization (DNP) experiments (Solmaz et al., 2020). Very recently, Hong and Sun (2021) have presented an NMR-on-a-chip transceiver that allows for phase-coherent detection at an arbitrary, non-zero intermediate frequency (IF) by using a dedicated design solution in the receiver path.

In this paper, we present a portable NMR system based on one of our NMR-on-a-chip transceiver application-specific integrated circuits (ASICs) with augmented functionalities. More specifically, the proposed NMR system provides the possibility of arbitrary phase modulation of the excitation pulse and, even for the case where the receiver local oscillator frequency is different from the excitation frequency, phase-coherent detection of the resulting NMR signal at a non-zero IF by the use of two commercially available direct digital synthesis (DDS) chips. Additionally, the presented system features an active, field-locking-based temperature compensation enabled by the DDS frequency synthesizers (Issadore et al., 2011; Lei et al., 2017).

The paper is organized as follows. Section 2 discusses some key design considerations of portable, low-field NMR platforms and introduces the proposed system architecture. Section 3 then describes the utilized transceiver chip and DDS-based reference signal generator as well as the signal processing unit and the utilized probe head. Finally, Sect. 4 provides measurement results of the proposed system that verify its functionality before the paper closes with a discussion and a brief outlook on future work in Sect. 5.

## 2 Design consideration and system architecture

Before introducing the proposed system architecture, we will discuss some critical design considerations of portable NMR detection systems. In contrast to high-field NMR inside superconducting magnets, low-field NMR based on permanent magnets and NMR-on-a-chip transceivers suffers from a low signal-to-noise ratio (SNR) and magnetic field drift. The former is mainly due to the low polarization levels, the low operating frequency, and the limited transmitter power of NMR-on-a-chip transceivers. The latter is due to the large temperature coefficient of standard permanent magnet materials (up to 1200 ppm K ${}^{-1}$ for NdFeB magnets and 500 ppm K ${}^{-1}$ for SmCo magnets).

### 2.1 Phase control

According to the reciprocity principle, the SNR of the NMR signal in the time domainIgnoring the inhomogeneity of the $B_{0}$ and $B_{1}$ field over the sample volume. can be written as (Hoult and Richards, 1976; Hoult, 2000)

1 $${\mathrm{SNR}}_{t}=\frac{\omega_{0}B_{u}M_{0}V_{s}}{\sqrt{4kT\Delta fR_{\mathrm{coil}}}},$$

where $\omega_{0}$ is the nuclear Larmor frequency ( $\omega_{0}=\gamma B_{0}$ ), $B_{u}$ is the unitary magnetic field of the detection coil, $M_{0}\propto \omega_{0}$ is the nuclear magnetization, $V_{s}$ is the sample volume, and $R_{\mathrm{coil}}$ is the AC coil resistance, which is proportional to the square root of the operating frequency due to the skin effect without considering the proximity effect (Minard and Wind, 2001). k is Boltzmann's constant, T is absolute temperature, and Δf is the considered detection bandwidth. Thus, SNR decreases rapidly as the working frequency decreases $\left(\mathrm{SNR}\propto \omega_{0}^{7/4}\right)$ , which is one of the main limitations of low-field NMR.

**Figure 1:**
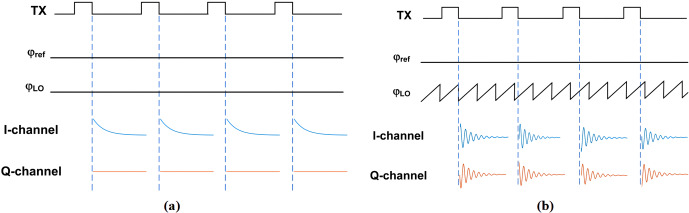
Illustration of the effect of the choice of the receiver LO frequency on the phase of the down-converted NMR signal. **(a)** Homodyne detection and **(b)** low-IF detection.

One standard method to improve the SNR is to repeat the NMR experiments many times and average the individual results in the time domain, introducing a strong trade-off between signal quality (SNR) and measurement time. Importantly, for time-domain averaging to work properly, the signal phase has to be constant across all individual NMR time traces and different excitation pulse lengths. In the following, we will refer to this property as “phase-coherent detection”. Today, most modern NMR spectrometers make use of quadrature detection (Keeler, 2013), allowing for both a receiver local oscillator (LO) frequency that equals the excitation frequency (homodyne detection) as well as a (small) offset between the excitation and the receiver local oscillator frequencies (low-IF detection) (Bürkle et al., 2020; Issadore et al., 2011; Hong and Sun, 2021). The former benefits from the intrinsically coherent output phase across different scans but introduces a trade-off between excitation efficiency (on-resonance vs. off-resonance excitation) and 1/f noise (for a single spectral line, on-resonance excitation results in zero IF, while off-resonance excitation produces an intrinsic non-zero IF). The latter allows for both on-resonance excitation and a non-zero IF to alleviate the influence of 1/f noise on the overall SNR. However, the low-IF approach also comes at the expense of an uncertain receiver phase if no dedicated countermeasures are taken. This is illustrated in Fig. 1. Here, we have, without loss of generality, assumed a simple sample containing a single homogeneously broadened line at a Larmor frequency of $f_{L}$ . In this case, on-resonance excitation corresponds to $f_{\mathrm{TX}}=f_{L}$ , where $f_{\mathrm{TX}}$ is the frequency of the excitation pulse. Under these conditions, the rotating frame of reference during the pulse and during detection of the NMR signal after the pulse rotates with the same frequency of $f_{L}=f_{\mathrm{TX}}$ . As illustrated in the figure, when the frequency of the local oscillator in the receiver, $f_{\mathrm{LO}}$ , is equal to the frequency of the rotating frame of reference, i.e., $f_{\mathrm{LO}}=f_{\mathrm{TX}}=f_{L}$ , the phase of the downconverted NMR signal is constant. By contrast, if the local oscillator frequency is different from the frequency of the rotating frame of reference, i.e., $f_{\mathrm{LO}}\neq f_{\mathrm{TX},L}$ , the downconverted NMR signal has a non-constant phase that depends on the offset frequency of the rotating frame of reference, $f_{\mathrm{TX}}=f_{L}=f_{\mathrm{TX},L}$ , and the LO frequency, $f_{\mathrm{LO}}$ , according to $\Delta f=f_{\mathrm{IF}}=f_{\mathrm{TX},L}-f_{\mathrm{LO}}$ , which is the inter-pulse distance and the pulse duration. Intuitively, for Δf≠0 , the receiver observes the NMR signal at the wrong angular velocity, leading to a residual rotation of the NMR signal after down-conversion, which, in turn, leads to the non-constant phase of the downconverted NMR signal.

Recently, Hong and Sun (2021) proposed a custom-designed hardware solution for the phase coherence problem in low-IF receivers explained above. Their solution is based on delaying the transmit signal generated by the pulse controller until the phase of the excitation pulse is in phase with the LO signal in the receiver, resulting in a constant phase of the downconverted NMR signal across multiple scans. This is achieved by the use of a phase alignment detector (a phase-frequency detector followed by a resistor–capacitor (RC) low-pass filter and an inverting Schmitt trigger), which delays the software transmit signal from the pulse controller until the phase of the excitation pulse is in phase with the receiver LO signal. While this scheme works well for pulse schemes with fixed inter-pulse delays such as the CPMG (Carr–Purcell–Meiboom–Gill) sequences presented in Hong and Sun (2021), it is not suitable for experiments that require variable, arbitrarily and precisely definable time intervals between consecutive excitation pulses such as inversion recovery (IR) experiments. More specifically, the phase alignment circuit proposed in Hong and Sun (2021) introduces a non-constant delay, $T_{\mathrm{delay}}$ , between the software transmit signal from the pulse controller and the actual transmit pulse at the output of the hardware alignment circuit, which can be as large as $T_{\mathrm{delay}}\leq 1/f_{\mathrm{IF}}$ , where $1/f_{\mathrm{IF}}$ is the period corresponding to the utilized IF frequency. As an example, the IF of $f_{\mathrm{IF}}=50$ kHz used in Hong and Sun (2021) introduces an uncertainty in the actual pulse occurrence of up to 20 µ s. To avoid this problem, it has to be possible to synchronize the receiver LO signal to the transmitter waveform at will without introducing non-constant delays in the transmitter timing.

Moreover, the phase of the excitation pulse should also be adjustable at will to enlarge the range of possible pulse sequences, including, for example, phase cycling. Phase cycling is a very useful capability for low-field NMR-on-a-chip platforms, because it mitigates the effect of gain and phase mismatch between the in-phase and quadrature outputs of the on-chip and PCB-based electronics without the need for (expensive) calibration. Without proper countermeasures, the gain and phase mismatch cause an imperfect cancelation of the image frequency, i.e., the frequency at $f_{\mathrm{IM}}=f_{\mathrm{LO}}\pm f_{\mathrm{IF}}$ , when the desired signal is located at $f_{\mathrm{NMR}}=f_{\mathrm{LO}}\mp f_{\mathrm{IF}}$ , distorting the NMR spectrum. Phase cycling is an effective method to remove these artifacts (Rahman et al., 2016), which requires arbitrary phase control of the transmitter phase and, if a non-zero IF is used, measures to ensure a coherent phase of the receiver LO signal.

In this paper, we propose a solution for the abovementioned problem based on two commercially available DDS chips that allows for an arbitrary intermediate frequency, an arbitrary phase of the excitation pulse, and a phase-coherent receiver LO signal without compromising the pulse timing. The details of the proposed scheme are given in Sect. 3.2.

### 2.2 Temperature drift

In most state-of-the-art low-field NMR platforms, permanent magnets are used due to their small size and low power consumption (Alnajjar et al., 2021; Yang et al., 2021). One limitation of such systems is that temperature fluctuations can cause severe frequency drifts if no countermeasures are taken. For instance, considering a free induction decay (FID) signal with a full width at half maximum (FWHM) of 100 Hz in the frequency domain measured with a 0.5 T NdFeB permanent magnet, the temperature would need to be kept constant with a precision of around 0.004 ${}^{\circ }$ C to avoid artifacts in the spectrum. Fortunately, various methods can be used to stabilize the NMR spectrum against environmental temperature fluctuations. For example, keeping the magnet in a temperature-controlled box is the most common method (Yu et al., 2018). However, this approach requires the temperature control of a large volume and is, therefore, quite power hungry. Actively adjusting the static $B_{0}$ field by means of a field-frequency lock (FFL) is another common method to stabilize the field over time. This approach uses a feedback control based on real-time measurements of an NMR reference signal – frequently of a nucleus other than the one under observation in the main channel – in an auxiliary NMR channel that is operated in parallel with the main channel (Hoult et al., 1978; Kan et al., 1978; Chen et al., 2018). With this method, the magnetic field can be stabilized at sub-ppm levels of accuracy over several hours of measurement time (Takahashi et al., 2012). Alternatively, a high-precision Hall sensor can also be used to monitor the $B_{0}$ field over time (Lei et al., 2017). One drawback of the conventional FFL method is that it requires a dedicated lock channel, significantly increasing the hardware complexity, especially for low-cost portable NMR systems. As an alternative to adjusting the $B_{0}$ field, the feedback loop in the FFL method can also be closed by modifying the excitation frequency to follow the measured changes in the Larmor frequency (Issadore et al., 2011; Lei et al., 2015). Here, it should be noted that all FFL-based methods that use a single field probe to measure the $B_{0}$ field can only be used to control the average $B_{0}$ field but cannot compensate for temperature-induced changes of the field homogeneity, which can largely deteriorate the achievable frequency resolution when averaging over a long time. Therefore, several signal processing techniques based on the measured NMR signal can be used to complement the abovementioned hardware measures to improve system robustness against temperature fluctuations further (Morris et al., 1997; Ha et al., 2014). Among them, a compensation method based on reference deconvolution is widely used to obtain a high-resolution NMR spectrum in the presence of spatially fluctuating $B_{0}$ fields (Morris, 1988; Barjat et al., 1995; Iijima and Takegoshi, 2008). In this approach, the ideal spectrum is reconstructed by deconvoluting the measured (main) NMR spectrum with the ratio of the measured and the ideal reference signals.

In this paper, we propose a field-locking-based solution for eliminating the effect of magnet temperature drift on the measurement results that uses a digital control loop to adjust the excitation frequency automatically based on the measured IF. The details of the proposed method are also given in Sect. 3.2.

### 2.3 Proposed system architecture

**Figure 2:**
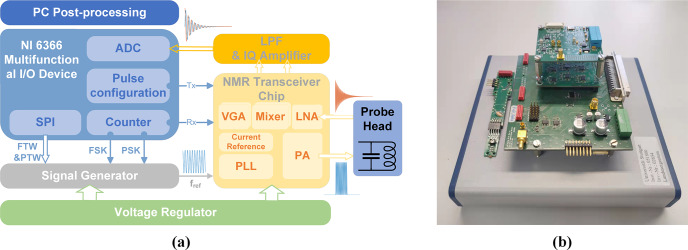
**(a)** Block diagram and **(b)** photograph of all electronics of the presented CMOS-based NMR platform.

The architecture of the proposed portable NMR system is shown in Fig. 2. It comprises five main building blocks: (i) an RF coil for sample excitation and detection of the NMR signal, (ii) a CMOS NMR-on-a-chip transceiver containing all performance-critical analog transceiver electronics, (iii) a reference signal generator to generate frequency- and phase-adjustable excitation pulses and a phase-coherent receiver LO signal, (iv) a digital signal processing unit for system control and signal acquisition, and (v) a motherboard that integrates all required power management and further signal conditioning electronics such as anti-aliasing filters and level shifters.

## 3 System implementation

### 3.1 NMR-on-a-chip transceiver and signal conditioning electronics

**Figure 3:**
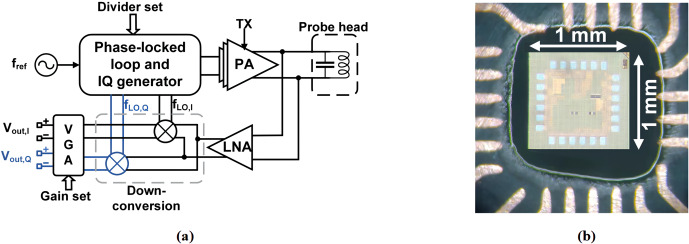
**(a)** Block diagram of the custom CMOS transceiver chip. **(b)** A micrograph of the chip attached to the PCB-based NMR-probe head.

According to Fig. 3a, the custom-designed CMOS NMR-on-a-chip transceiver consists of a programmable integer-N phase-locked loop (PLL), a quadrature (IQ) generator, a power amplifier (PA), a low noise amplifier (LNA), a quadrature down-conversion mixer, and two variable gain amplifiers (VGAs). Depending on the chip configuration, the on-chip PLL can be used to multiply the external reference frequency by a selectable scaling factor between 1 and 64. Alternatively, the PLL can be bypassed, and the external reference signal is divided by 2 to produce the required quadrature LO signals from the external reference. In the latter mode of operation, the chip can produce excitation frequencies down to DC, while the minimum detectable frequency in the receiver path is limited by an on-chip coupling capacitor between the LNA and the mixers. Since the on-chip PLL can operate with reference frequencies between 5.7 and 12.1 MHz, overall, the chip has an operating frequency range between 5 and 770 MHz. The H-bridge-based PA uses a separate supply voltage of 2.5 V and provides a maximum peak-to-peak output current of 180 mA into a 10 Ω load.

In the receiver path, the NMR signal is first amplified by an LNA and then down-converted by a quadrature mixer to an intermediate frequency (IF). Although zero-IF operation is possible, we typically operate the chip with an IF between 50 and 200 kHz to avoid SNR degradation due to flicker noise. The LNA features a state-of-the-art input-referred voltage noise of 770 pV Hz ${}^{-\frac{1}{2}}$ . The measured maximum combined gain of the LNA and the mixer is 45 dB. The two digitally programmable VGAs provide a gain between 0 and 40 dB. The two VGAs are followed by a pair of off-chip PCB-based fourth-order Bessel low-pass filters (LPFs) with a fixed gain of 28 dB and a fixed cutoff frequency at 350 kHz. The maximum overall gain of the receiver chain is 113 dB. Figure 3b shows a micrograph of the NMR-on-a-chip transceiver. The chip is implemented in a 130 nm SiGe BiCMOS technology offered by STMicroelectronics.

At this point, it is worth mentioning that the NMR-on-a-chip transceiver of Fig. 3a uses a single PLL with a single reference frequency to generate both the excitation and the local oscillator signal for the quadrature down-conversion mixer. In the following section, we will explain how it is still possible to provide both excitation pulses with an arbitrary phase and timing and a phase-coherent down-converted NMR signal at a non-zero IF.

### 3.2 Proposed phase-coherent reference signal generator with temperature compensation

**Figure 4:**
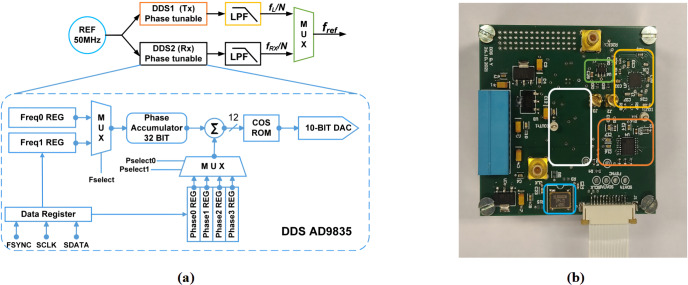
**(a)** Block diagram of the proposed DDS reference generator and **(b)** its PCB-based implementation on a 56 × 57 mm two-layer PCB. Corresponding blocks are highlighted with the same color in the block diagram and the PCB photograph. The white rectangle indicates the position of the second DDS module on the bottom layer of the PCB.

Direct digital synthesis (DDS) is a common choice for providing accurate reference signals in modern commercial NMR spectrometers, because it can conveniently and rapidly change the frequency, phase, and amplitude of a waveform. To allow for both phase coherence in the receiver path and phase adjustability of the excitation pulse, in this paper we propose a synchronous reference signal generator based on two commercially available DDS chips (AD9835, Analog Devices; AD9835, 2022), according to Fig. 4a. The two DDS chips (DDS1 and DDS2) utilize the same crystal oscillator (LFSPXO023414) with a clock frequency of 50 MHz to enable phase synchronicity between their two output signals. The 50 MHz reference frequency was selected to satisfy the Nyquist theorem with some margin for the largest NMR transceiver PLL reference frequency of 12.1 MHz. The AD9835 possesses two frequency registers, each of which can be programmed with a 32-bit frequency tuning word (FTW). A 1-bit frequency select input (FSELECT) then determines which FTW is used in the phase accumulator. Each AD9835 has two frequency registers that allow for fast switching between two different frequencies, e.g., to perform a frequency shift keying (FSK) modulation, where each frequency can be defined with a resolution of 11.6 mHz. Furthermore, there are four additional 12-bit phase tuning word (PTW) registers that allow for an arbitrary adjustment of the phase of the DDS output signal with a resolution of 1.53 mrad (0.088 ${}^{\circ }$ ). A 2-bit phase select input (PSEL0, PSEL1) determines which PTW is used for the phase accumulator. The output of each DDS chip is filtered and amplified by an active second-order Butterworth LPF with a cutoff frequency of 12.8 MHz and a gain of 6 dB. The current reference frequency for the CMOS transceiver, $f_{\mathrm{ref}}$ , is selected with an analog switch (TS5A63157, Texas Instruments, Inc.), which displays a maximum delay of 5 ns and an isolation of 61 dB.

**Figure 5:**
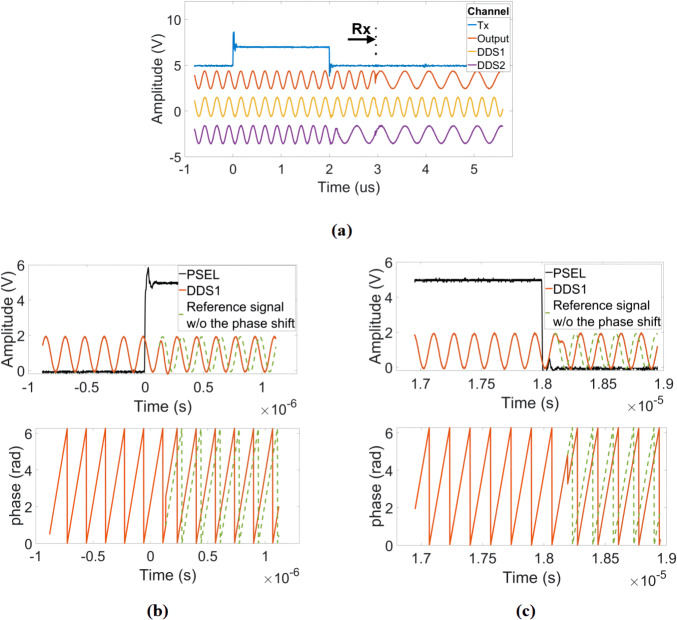
Illustration of the timing of the proposed DDS-based reference signal generator. **(a)** Frequency shift in the receiver DDS at the falling edge of the excitation pulse with a phase that is synchronous with the current transmitter phase. **(b, c)** Amplitude and phase response to ±90 ${}^{\circ }$ phase shifts at the rising edge and falling edge of the PSEL digital signal.

The detailed method to generate a reference frequency $f_{\mathrm{ref}}$ that allows for both an arbitrary adjustment of the phase of the excitation pulse and a phase-coherent receiver LO when operating at an arbitrary IF is as follows. First, the outputs of both DDS chips are deactivated, and their phase accumulators are set to zero. Then, two groups of FTWs are simultaneously loaded into the two frequency registers of each DDS, the first corresponding to the Larmor frequency of the nucleus of interest $f_{L}$ and the second to the desired receiver LO frequency ( $f_{\mathrm{RX}}=f_{L}+\Delta f)$ . Additionally, four PTWs $\left(0{}^{\circ },\frac{90{}^{\circ }}{N},\frac{180{}^{\circ }}{N},\frac{270{}^{\circ }}{N}\right)$ , with N being the divider ratio of the PLL of the CMOS transceiver chip, are also loaded into the four phase registers of each DDS. Here, it should be noted that the FTWs are initially selected such that both DDS chips start with the same output frequency of $f_{L}/N$ . Although, in this configuration, the reference frequency of the PLL corresponds to the excitation frequency, the transceiver chip is in its receive (RX) mode until the chip's transmit (TX) signal is enabled. Setting both DDS values to a value of $f_{L}/N$ ensures that the phase of the receiver DDS is synchronous with the transmitter phase when the frequency of the NMR-on-a-chip transceiver is changed to $f_{\mathrm{RX}}/N$ at the end of the transmitter pulse. We will explain this important point below in more detail. After that, both DDS outputs are activated. The digital control signal for the FSELECT port of DDS2 is triggered by the software transmit (TX) signal. More specifically, a falling edge of the transmit (TX) signal causes the output frequency of DDS2 to change from $f_{L}/N$ to $f_{\mathrm{RX}}/N$ with a continuous phase. In this way, it is ensured that the receiver LO phase is synchronized to the phase of the latest excitation pulse, even when using different excitation pulse phases. Since the multiplexer switch is not toggled from DDS1 to DDS2 until the end of the excitation pulse, the PLL reference frequency $f_{\mathrm{ref}}$ remains connected to the output of DDS1, which still operates at a frequency of $f_{L}/N$ , ensuring an excitation of the spin ensemble at a frequency of $f_{L}$ . After a delay of a few microseconds from the falling edge of the transmit (TX) signal, the new reference frequency $f_{\mathrm{ref}}=f_{\mathrm{RX}}/N$ is finally applied to the PLL of the NMR-on-a-chip transceiver, and the PLL locks to this new reference frequency and phase within a few microseconds. The PLL locking time is determined by its bandwidth of a few hundred kilohertz. Here, the on-chip PLL was designed to lock faster than the coil's/receiver's typical dead time. Overall, in this way, DDS1 is allowed to run continuously at a frequency of $f_{L}/N$ , preserving its phase, which is the key requirement for phase-coherent excitation pulses in multi-pulse NMR experiments, and DDS2 provides an LO signal for the NMR-on-a-chip transceiver that is coherent with the last excitation pulse. Figure 5a summarizes and illustrates the overall timing of the DDS output explained above for a single excitation pulse. The PSEL bit can be used to select between different phases for the excitation pulse, i.e., the output of DDS1, to perform, for example, phase cycling or generate a classical CPMG sequence. Figure 5b and c show how the PSEL bit is used to provide a phase change of ±90 ${}^{\circ }$ in the output of DDS1. Finally, it should be noted that, for multi-scan measurements with averaging, the phase accumulator must be set to zero after each scan.

In addition to providing a phase-coherent non-zero IF detection with an arbitrary excitation phase, the presented DDS-based frequency synthesizer can also be used to solve the temperature drift problem explained in Sect. 2.2. More specifically, to overcome this problem, our proposed NMR platform incorporates an automatic control loop, which automatically updates the FTW of each DDS to provide an on-resonance excitation at the Larmor frequency even in the presence of $B_{0}$ field drifts. The automatic control loop works as follows. After each excitation pulse, the frequency information is extracted from the acquired FID or spin-echo signal, respectively. This measured frequency is compared with the predefined (in the control software) intermediate frequency of a selected peak in the spectrum. The implemented algorithm then updates the FTW of both DDS1 and DDS2 to provide an on-resonance excitation and keep the measured IF constant.

### 3.3 Data acquisition and digital signal processing

For data acquisition and digital signal processing, we used a commercial multifunction I/O device (USB-6366, National Instruments). The USB-6366 offers eight differential 16-bit analog input ports with a sampling rate of 2 MS s ${}^{-1}$ per channel and 24 digital I/O channels. Our system uses an 8-bit digital output bus to generate the required digital control waveforms with a maximum sample frequency of 1 MHz, e.g., to communicate with the NMR-on-a-chip transceiver and the DDS chips via SPI. The USB-6366 also offers four 32-bit counters/timers, which can be used for pulse generation and event counting. To orchestrate the experiments, we have developed a custom-made LabVIEW-based NMR control software. The software controls the communication with each DDS and the programmable NMR-on-a-chip transceiver; the generation of the NMR sequences, including phase control; and the analysis of the acquired NMR signals.

### 3.4 NMR probe head

The NMR probe heads for the experiments presented in this paper consist of an NMR coil and a tuning capacitor without matching to benefit from the intrinsic noise-free preamplification of the inductor–capacitor (LC) resonator formed by the coil and the tuning capacitor (Anders et al., 2016; Handwerker et al., 2013). Impedance matching is not required due to the close spatial proximity of the LC resonator and the in-field NMR-on-a-chip transceiver.

**Figure 6:**
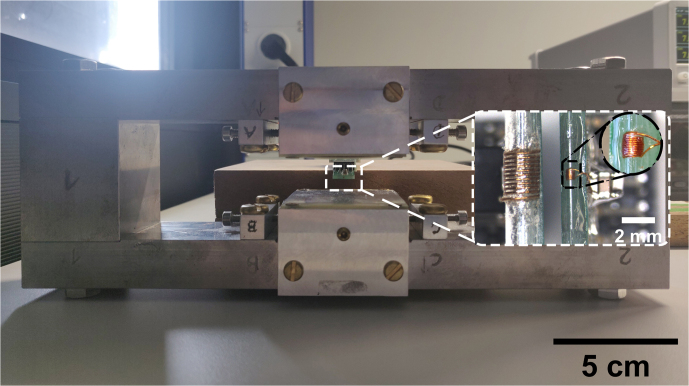
Photograph of the utilized 0.36 T magnet with the two NMR coils as inset. Left: the relaxometry probe. Right: the spectroscopy probe.

To demonstrate the versatility of the proposed NMR platform, we have used two different solenoidal coils with largely different sizes, see the inset of Fig. 6. The first coil (spectroscopy probe) is optimized for spin sensitivity. It is implemented as a 10-turn solenoidal coil by winding tightly – i.e., leaving a spacing as small as possible between adjacent turns – a 50 µ m enameled copper wire around a small-diameter glass capillary (i.d.: 0.38 mm, o.d.: 0.4 mm). At 62 MHz, the coil displays a measured impedance of ( 0.6+j13.4 ) Ω with an inductance of 34.25 nH. In combination with our NMR-on-a-chip transceiver, the spectroscopy probe produces 90 ${}^{\circ }$ pulse lengths of 5 µ s, corresponding to an effective $B_{1}$ field of 1.2 mT. The second coil (relaxometry probe) was optimized towards improved concentration sensitivity for relaxometry measurements, taking into account the limited driving strength of the utilized broadband NMR-on-a-chip transceiver. It is implemented as a 10-turn solenoid by winding a 0.12 mm enameled copper wire around a larger glass tube (i.d.: 1.5 mm, o.d.: 2 mm) using the bifilar winding method (Wu et al., 1994). At 15.3 MHz, the coil displays a measured impedance of ( 1.0+j15.0 ) Ω with an inductance of 156 nH. In combination with our NMR-on-a-chip transceiver, the relaxometry probe produces 90 and 180 ${}^{\circ }$ pulse lengths of 18 and 38 µ s, respectively. The latter value is deduced from the best echo quality. The 90 ${}^{\circ }$ pulse length corresponds to an effective $B_{1}$ field of 330 µ T.

Samples are inserted into the two coils by using capillary glass tubes with outer diameters of 0.3 and 1.3 mm.

For the following experiments, two different magnets with $B_{0}$ field strengths of 0.36 and 1.45 T were used. The first magnet ( $B_{0}=0.36$ T), which was used for the relaxometry measurements, is a custom-designed H-shaped magnet that features a small volume of 26×9.9×5 cm ${}^{3}$ , a relatively low weight, and a moderate homogeneity of 20 ppm over the sample volume. The second one ( $B_{0}=1.45$ T), which was used for the spectroscopy experiments below, is a stripped-down Bruker MiniSpec magnet, which provides better homogeneity at a larger form factor and weight.

## 4 Experimental results

In order to verify the performance of the proposed NMR platform, we have conducted a series of experiments. Here, we have first verified the phase coherence between multiple acquisitions in multi-scan experiments for efficient time-domain averaging. To this end, we compared the phase distribution of the actual FID signals when using different reference signal sources for the NMR transceiver chip. More specifically, a commercial waveform generator (Keysight 33600A) equipped with a frequency shift keying (FSK) option was used as an alternative reference signal source for our proposed DDS-based signal generator. The use of FSK for phase-coherent averaging in combination with NMR-on-a-chip transceivers and non-zero IFs was first proposed in Handwerker et al. (2020). This approach is sufficient for phase-coherent averaging in simple pulse-acquire experiments, because all standard FSK implementations switch the frequency phase coherently (typically at the zero crossings of the phase). However, the FSK approach does not allow for coherent control of the phase of the excitation pulse across multiple pulses. In these experiments, we compared both on-resonance, i.e., $f_{\mathrm{TX}}=f_{L}$ , and off-resonance, i.e., $f_{\mathrm{TX}}\neq f_{L}$ , excitation. Here, off-resonance excitation allows for a non-zero IF of $f_{\mathrm{IF}}=f_{\mathrm{TX}}-f_{L}$ without the need to switch the reference frequency between transmit (TX) and receive (RX) at the expense of reduced excitation efficiency. To obtain statistics on the phase distribution of the individual FIDs, we recorded 10 consecutive FIDs for the commercial frequency generator and the proposed DDS-based solution using the relaxation probe filled with a vegetable oil sample.

The detailed steps of calculating the phase distributions are as follows. The original FID signal length was first extended by a factor of 3 using zero padding. Then, we Fourier transformed the extended FIDs and adjusted the phase to obtain a pure absorption spectrum in the real part of the spectrum. Since there was only one line in the spectrum, it was sufficient to use a zero-order phase correction with a single correction phase

$\phi_{\mathrm{FID}}=-\phi_{\mathrm{corr}}$



$\phi_{\mathrm{corr},i}$ (Keeler, 2013) for each FID. We then computed the standard deviation of the correction phases of all 10 FIDs $\phi_{\mathrm{corr},i}$ . The results are listed in Table 1. The data clearly show that the proposed DDS solution allows for phase-coherent time-domain averaging with a performance that is on par with a medium-priced commercial instrument.

**Table 1:** Comparison of the phase distribution of the recorded FID signals for the proposed DDS-based frequency generator and commercial instruments for 10 consecutive scans.

	Off-resonance	On-resonance	On-resonance
	( $f_{\mathrm{TX}}\neq f_{L}$ )	( $f_{\mathrm{TX}}=f_{L}$ )	( $f_{\mathrm{TX}}=f_{L}$ )
	(33600A)	(33600A)	(DDS)
Standard	0.27 ${}^{\circ }$	0.34 ${}^{\circ }$	0.17 ${}^{\circ }$
deviation $\sigma_{\phi_{\mathrm{corr},i}}$			

**Table 2:** Phase distribution and SNR of the recorded FID signals using the proposed DDS-based frequency generator for up to 4000 scans.

Number of scans, $N_{\mathrm{scans}}$	500	1000	2000	3000	4000
Standard deviation, $\sigma_{\phi_{\mathrm{corr},i}}$	0.43 ${}^{\circ }$	0.52 ${}^{\circ }$	0.68 ${}^{\circ }$	0.70 ${}^{\circ }$	0.76 ${}^{\circ }$
Frequency drift	+3 kHz	+11 kHz	- 9 kHz	+2 kHz	- 4 kHz
Mean, $\mu_{\mathrm{SNR}}$	18.4	18.2	18	18	17.8
Enhanced SNR	401	554	769	929.7	1046
Enhancement	21.8	30.4	42.7	51.7	58.8

**Figure 7:**
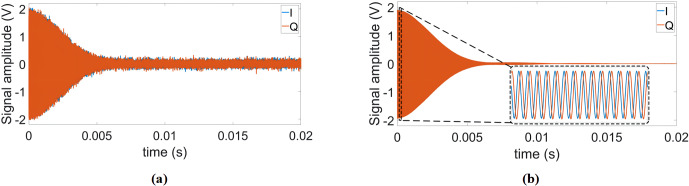
**(a)** Single scan and **(b)** time-averaged ( $N_{\mathrm{scans}}=4000$ ) FID signals using our proposed DDS-based reference signal generator.

Additionally, to test the system stability during a long-term averaging measurement, we also recorded 4000 consecutive FID signals over 4 h. In these experiments, the proposed field-locking-based temperature compensation scheme was enabled. Table 2 summarizes the results of this experiment. According to the table, there is a small (below 1 ${}^{\circ }$ ) increase in the standard deviation of the measured correction phase over time. We attribute this to mainly two effects: first, the adjustment of the excitation frequency sometimes cannot keep up with the temperature drift rate, and, second, there is a drift of the field homogeneity vs. time. Importantly, the improvement factor due to averaging is close to the theoretically predicted value of $\sqrt{N_{\mathrm{scans}}}$ . Figure 7 shows the single scan and time-averaged FID signals using the proposed DDS-based signal generator, an IF of 100 kHz, and a repetition time of $T_{R}=1.5$ s.

**Figure 8:**
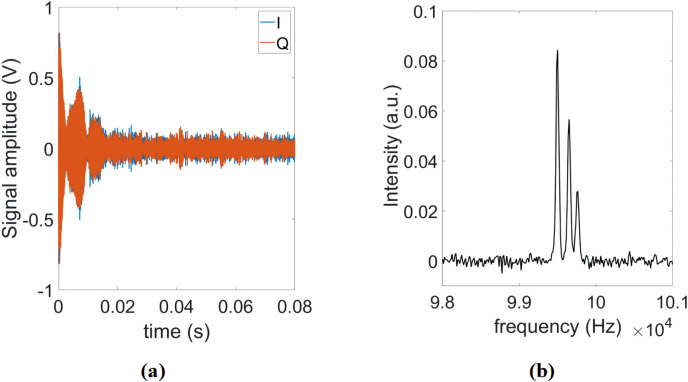
**(a)** Single-shot FID of ethanol. **(b)** Real part of the corresponding Fourier spectrum.

Next, the FID of a 21.2 nL pure ethanol sample was measured using the spectroscopy probe (see Fig. 8a). Figure 8b shows the real part of the corresponding Fourier spectrum. The position and amplitude of the three peaks in the spectrum correspond to the expected chemical shifts and numbers of hydrogen nuclei in the hydroxyl (OH), methylene (CH ${}_{2}$ ), the methyl (CH ${}_{3}$ ) groups. Considering the time-domain SNR of the ethanol sample, the calculated time-domain spin sensitivity is $3.2\times 10^{16}$ spins Hz ${}^{-\frac{1}{2}}$ (Anders et al., 2009).

**Figure 9:**
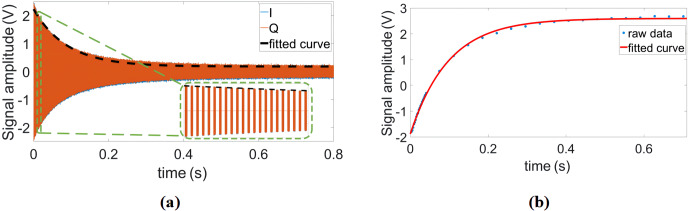
**(a)** CPMG signal and **(b)** IR signal of sunflower oil.

Having confirmed the functionality of the proposed scheme for coherent excitation and detection, we proceeded by measuring transverse relaxation ( $T_{2}$ ) and longitudinal relaxation ( $T_{1}$ ) times of sunflower oil, as an example of a homogeneous sample, with conventional CPMG and IR-FID sequences. The $T_{2}$ time was extracted with the following parameters for the CPMG sequence: number of echoes NE = 800, echo time TE = 1 ms, and an echo duration of 0.6 ms. The $T_{1}$ time was extracted with the following parameters for the IR-FID sequence: a minimum delay of 3 ms, a maximum delay of 701 ms, and a number of steps of 38. Here, the phase information of each FID (or echo when using IR-echo sequence) can be used to distinguish the sign of those signals whose amplitudes are close to zero. Relying on a variable pulse delay between the first 180 ${}^{\circ }$ pulse and the first 90 ${}^{\circ }$ pulse, the IR sequence requires precise timing of said two pulses and a phase-coherent detection of the FID or the echo. Therefore, the IR experiments for $T_{1}$ extraction serve as an excellent benchmark application for the proposed DDS-based reference generator. Example data of the CPMG and the IR measurements are shown in Fig. 9. The corresponding relaxation times derived from single exponential fitting were $T_{2}=89$ ms and $T_{1}=95.4$ ms, respectively.

**Figure 10:**
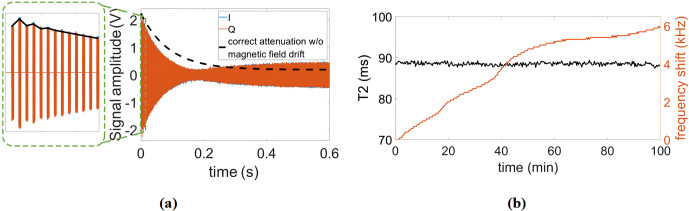
**(a)** Illustration of the effect of temperature-induced magnetic field drifts on the CPMG signal. **(b)** Continuously measured $T_{2}$ and the frequency drift of the magnet during the measurement time.

Having verified the functionality of the proposed reference signal generator, we also tested the performance of the proposed field-locking-based temperature compensation scheme in relaxometry measurements. Frequency shifts caused by temperature fluctuations not only reduce frequency-domain resolution (see Sect. 2.2) but also have a significant influence on the accuracy of relaxation time measurements. More specifically, if uncompensated, the fixed excitation frequency will gradually deviate from the Larmor frequency over time. In this case, the $B_{0}$ field does not completely disappear in the rotating frame, and the effective rotation axis is therefore tilted out of the xy -plane (Korzhnev et al., 2000). In addition, due to a less efficient off-resonance excitation with increasing offset frequencies caused by the frequency drift, the predefined duration of the π pulses might no longer be able to flip the magnetization by 180 ${}^{\circ }$ , resulting in distorted CPMG signals. Here, two types of distortion are commonly encountered. The first one is an oscillation of the first few points of the CPMG signal (see Fig. 10a, left), and the second is an incomplete decay of the echo signal (see Fig. 10a, right). It should be mentioned that the correct attenuation line (dashed line in the figure) was obtained from a measurement with the field-locking-based temperature compensation scheme enabled. Figure 10b shows a series of CPMG experiments for a continuous measurement of the transverse relaxation time ( $T_{2}$ ) over a total experimental time of nearly 100 min without temperature control of the magnet. According to the figure, the Larmor frequency drifted over almost 6 kHz, but thanks to the frequency control loop, the $T_{2}$ time was still extracted with high accuracy. More specifically, the mean and standard deviation of all recorded $T_{2}$ values in Fig. 10b are 88.5 and 0.4 ms, respectively, corresponding to a normalized standard deviation of 0.45 %.

**Figure 11:**
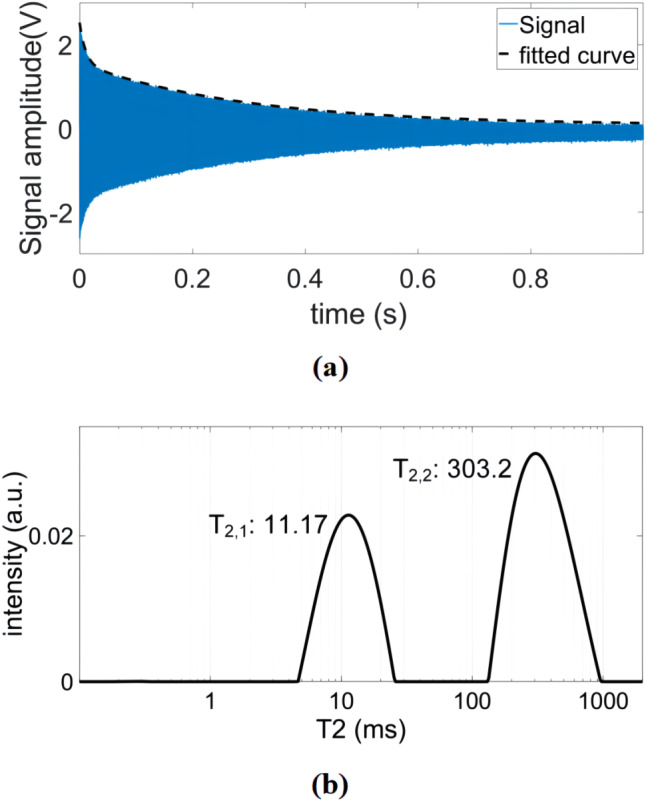
**(a)** An example time-domain CPMG signal of the constructed sample containing solutions with two different concentrations of copper sulfate doped water (5 and 75 mM). **(b)** The corresponding $T_{2}$ distribution after inverse Laplace transform.

To demonstrate that the presented platform is also capable of measuring the relaxation times of heterogeneous samples that contain materials with different relaxation times, we have analyzed samples containing copper sulfate solutions with two different concentrations per sample with concentrations ranging from 5 to 75 mM. These samples were used to emulate bound and unbound water samples since their distinction is a current application of NMR relaxometry (Stapf, 2010; Wu et al., 2021). More specifically, we inserted the higher concentration sample into a 0.8 mm capillary, which was then inserted into a 1.3 mm capillary filled with a lower doped copper sulfate solution to construct a sample with two distinct relaxation times. Figure 11a shows an example time-domain CPMG signal of such a sample. From the time domain data, we have extracted the relaxation times using double exponential fitting and cross-checked these results with the inverse Laplace transform data (see Fig. 11b), achieving an excellent matching between the two methods. The extracted $T_{2}$ and $T_{1}$ values are summarized in Table 3. In these experiments, the CPMG sequence parameters were the following: number of echoes NE = 2500, echo time TE = 0.4 ms, and an echo duration of 0.2 ms. The IR sequence parameters were the following: a minimum delay of 1 ms, a maximum delay of 801 ms (5 mM), 401 ms (10 mM), 201 ms (25 mM), and 81 ms ( ≥50 mM), and a number of steps of 25.

**Table 3:** Relaxation times of different concentrations of copper sulfate solution.

Concentration	$T_{2}$	$T_{1}$
(mM)	(ms)	(ms)
5	300 ± 1.8	333 ± 6.3
10	84 ± 1.1	93 ± 1.0
25	34 ± 0.7	42 ± 0.8
50	16 ± 0.3	18 ± 0.3
75	11 ± 0.1	14 ± 0.3
100	8 ± 0.1	10 ± 0.4

## 5 Conclusion and discussion

In this paper, we have presented a CMOS-based NMR platform featuring arbitrary phase control and coherent detection in a non-zero IF receiver architecture as well as active automatic temperature compensation. The proposed platform is centered around a custom-designed NMR-on-a-chip transceiver. Thanks to the on-chip broadband PLL, our system can operate between 5 and 770 MHz. As one of the main innovations, the presented system features a DDS-based reference signal generator for the on-chip PLL that enables precisely timed excitation pulses with an adjustable phase and – at the same time – phase-coherent detection at a non-zero IF. The proposed system achieves a phase stability well below 1 ${}^{\circ }$ in consecutive pulse acquire experiments, which is on par with commercial equipment. NMR spectroscopy and relaxometry experiments inside 1.45 and 0.36 T permanent magnets verified the versatility and excellent performance of the presented platform. Moreover, the proposed NMR platform includes an automatic control loop that effectively counteracts frequency changes due to thermal drifts of the utilized permanent magnet. The efficiency of the frequency control loop has been verified by $T_{2}$ measurements over 100 min, producing a normalized standard deviation in the measured $T_{2}$ values of 0.45 % in the presence of significant temperature fluctuations. Here, we would like to point out that, despite its overall good performance and usefulness for stabilizing the average $B_{0}$ field, the presented field-locking-based temperature compensation scheme cannot compensate for changes in the field homogeneity over time, which can lead to reduced frequency resolution in the averaged signal. Moreover, the proposed approach relies on sufficient SNR in a single-shot experiment to extract the current Larmor frequency with enough precision. Finally, the update rate for the estimation of the Larmor frequency is limited by the experiment's repetition rate, potentially leading to limitations in the presence of relatively fast temperature fluctuations. This being said, compared to prior state of the art, the architecture of the presented scheme is less complex, and it can be easily implemented in the digital domain together with the proposed DDS-based frequency synthesizer, rendering it a very suitable solution for CMOS-based NMR platforms. The total peak power of all custom-designed electronics is 2.9 W, which allows for battery operation for several hours from a modern power bank. In the future, we will extend the presented NMR platform by the possibility of performing Overhauser dynamic nuclear polarization (ODNP) using our recent EPR-on-a-chip transceivers (Chu et al., 2018; Hassan et al., 2021) for enhanced sensitivity in order to open up new application scenarios for portable NMR systems.

## Data Availability

Code and data are available upon request.

## References

[bib1.bib1] (2022). https://www.analog.com/en/products/ad9835.html#product-overview.

[bib1.bib2] Alnajjar BMK, Buchau A, Baumgartner L, Anders J (2021). NMR magnets for portable applications using 3D printed materials. J Magn Reson.

[bib1.bib3] Anders J, Boero G (2008). A Low-Noise CMOS Receiver Frontend for MRI.

[bib1.bib4] Anders J, Lips K (2019). MR to go. J Magn Reson.

[bib1.bib5] Anders J, Chiaramonte G, SanGiorgio P, Boero G (2009). A single-chip array of NMR receivers. J Magn Reson.

[bib1.bib6] Anders J, SanGiorgio P, Boero G (2011). A fully integrated IQ-receiver for NMR microscopy. J Magn Reson.

[bib1.bib7] Anders J, Handwerker J, Ortmanns M, Boero G (2016). A low-power high-sensitivity single-chip receiver for NMR microscopy. J Magn Reson.

[bib1.bib8] Anders J, Dreyer F, Kruger D, Schwartz I, Plenio MB, Jelezko F (2021). Progress in miniaturization and low-field nuclear magnetic resonance. J Magn Reson.

[bib1.bib9] Barjat H, Morris GA, Swanson AG, Smart S, Williams SCR (1995). Reference Deconvolution Using Multiplet Reference Signals. J Magn Reson Ser A.

[bib1.bib10] Boero G, de Raad Iseli C, Besse PA, Popovic RS (1998). An NMR magnetometer with planar microcoils and integrated electronics for signal detection and amplification. Sensor Actuat A-Phys.

[bib1.bib11] Boero G, Frounchi J, Furrer B, Besse PA, Popovic RS (2001). Fully integrated probe for proton nuclear magnetic resonance magnetometry. Rev Sci Instrum.

[bib1.bib12] Bürkle H, Schmid K, Klotz T, Krapf R, Anders J (2020). A high voltage CMOS transceiver for low-field NMR with a maximum output current of 1.4 App.

[bib1.bib13] Bürkle H, Klotz T, Krapf R, Anders J (2021). A 0.1 MHz to 200 MHz high-voltage CMOS transceiver for portable NMR systems with a maximum output current of 2.0 App.

[bib1.bib14] Chen SS, Xu LY, Wang HZ, Dai SG (2018). Field-frequency lock approach for 21.3-MHz high-performance NMR relaxation analyzer. Aip Advances.

[bib1.bib15] Chen Y, Jiang XW, Wang JN, Wu ZX, Wu YC, Ni ZH, Yi H, Lu RS (2021). Sensitive Oxidation of Sorbitol-Mediated Fe
${}^{2+}$
 by H
${}_{2}$
O
${}_{2}$
: A Reliable TD-NMR Method for Clinical Blood Glucose Detection. Anal Chem.

[bib1.bib16] Chu A, Schlecker B, Lips K, Ortmanns M, Anders J (2018). An 8-Channel 13 GHz ESR-on-a-Chip Injection-locked VCO-array achieving 200 
µ
M-Concentration Sensitivity.

[bib1.bib17] Colnago LA, Wiesman Z, Pages G, Musse M, Monaretto T, Windt CW, Rondeau-Mouro C (2021). Low field, time domain NMR in the agriculture and agrifood sectors: An overview of applications in plants, foods and biofuels. J Magn Reson.

[bib1.bib18] Gan ZH, Hung I, Wang XL, Paulino J, Wu G, Litvak IM, Gor'kov PL, Brey WW, Lendi P, Schiano JL, Bird MD, Dixon LR, Toth J, Boebinger GS, Cross TA (2017). NMR spectroscopy up to 35.2 T using a series-connected hybrid magnet. J Magn Reson.

[bib1.bib19] Grisi M, Gualco G, Boero G (2015). A broadband single-chip transceiver for multi-nuclear NMR probes. Rev Sci Instrum.

[bib1.bib20] Grisi M, Vincent F, Volpe B, Guidetti R, Harris N, Beck A, Boero G (2017). NMR spectroscopy of single sub-nL ova with inductive ultra-compact single-chip probes. Scientific Reports.

[bib1.bib21] Ha D, Paulsen J, Sun N, Song YQ, Ham D (2014). Scalable NMR spectroscopy with semiconductor chips. P Natl Acad Sci USA.

[bib1.bib22] Handwerker J, Ortmanns M, Anders J, Eschelbach M, Chang P, Henning A, IEEE, Ham D (2013). An Active TX/RX NMR Probe for Real-Time Monitoring of MRI Field Imperfections.

[bib1.bib23] Handwerker J, Eder M, Tibiletti M, Rasche V, Scheffler K, Becker J, Ortmanns M, Anders J (2016). An Array of Fully-Integrated Quadrature TX/RX NMR Field Probes for MRI Trajectory Mapping.

[bib1.bib24] Handwerker J, Perez-Rodas M, Beyerlein M, Vincent F, Beck A, Freytag N, Yu X, Pohmann R, Anders J, Scheffler K (2020). A CMOS NMR needle for probing brain physiology with high spatial and temporal resolution. Nat Methods.

[bib1.bib25] Hassan MA, Elrifai T, Sakr A, Kern M, Lips K, Anders J (2021). A 14-channel 7 GHz VCO-based EPR-on-a-chip sensor with rapid scan capabilities.

[bib1.bib26] Hong SJ, Sun N (2021). Portable CMOS NMR System With 50-kHz IF, 10-
µ
s Dead Time, and Frequency Tracking. IEEE T Circuits I.

[bib1.bib27] Hoult DI (2000). The principle of reciprocity in signal strength calculations – A mathematical guide. Concept Magnetic Res.

[bib1.bib28] Hoult DI, Richards RE (1976). Signal-to-Noise Ratio of Nuclear Magnetic-Resonance Experiment. J Magn Reson.

[bib1.bib29] Hoult DI, Richards RE, Styles P (1978). Novel Field-Frequency Lock for a Superconducting Spectrometer. J Magn Reson.

[bib1.bib30] Iijima T, Takegoshi K (2008). Compensation of effect of field instability by reference deconvolution with phase reconstruction. J Magn Reson.

[bib1.bib31] Issadore D, Min C, Liong M, Chung J, Weissleder R, Lee H (2011). Miniature magnetic resonance system for point-of-care diagnostics. Lab Chip.

[bib1.bib32] Kan S, Gonord P, Fan M, Sauzade M, Courtieu J (1978). Automatic Nmr Field-Frequency Lock-Pulsed Phase Locked Loop Approach. Rev Sci Instrum.

[bib1.bib33] Keeler J (2013). Understanding NMR Spectroscopy.

[bib1.bib34] Kim J, Hammer B, Harjani R (2010). A Low Power CMOS Receiver for a Tissue Monitoring NMR Spectrometer.

[bib1.bib35] Kim J, Hammer B, Harjani R (2012). A 5-300MHz CMOS Transceiver for Multi-Nuclear NMR Spectroscopy.

[bib1.bib36] Korzhnev DM, Tischenko EV, Arseniev AS (2000). Off-resonance effects in N-15 T-2 CPMG measurements. J Biomol NMR.

[bib1.bib37] Lee H, Sun E, Ham D, Weissleder R (2008). Chip-NMR biosensor for detection and molecular analysis of cells. Nat Med.

[bib1.bib38] Lei K-M, Mak P-I, Law M-K, Martins RP (2015). A palm-size mu NMR relaxometer using a digital microfluidic device and a semiconductor transceiver for chemical/biological diagnosis. Analyst.

[bib1.bib39] Lei KM, Mak PI, Law MK, Martins RP (2016). A mu NMR CMOS Transceiver Using a Butterfly-Coil Input for Integration With a Digital Microfluidic Device Inside a Portable Magnet. IEEE J Solid-St Circ.

[bib1.bib40] Lei KM, Heidari H, Mak PI, Law MK, Maloberti F, Martins RP (2016). A Handheld 50pM-Sensitivity Micro-NMR CMOS Platform with B-Field Stabilization for Multi-Type Biological/Chemical Assays.

[bib1.bib41] Lei KM, Heidari H, Mak PI, Law MK, Maloberti F, Martins RP (2017). A Handheld High-Sensitivity Micro-NMR CMOS Platform With B-Field Stabilization for Multi-Type Biological/Chemical Assays. IEEE J Solid-St Circ.

[bib1.bib42] Lei KM, Ha D, Song YQ, Westervelt RM, Martins R, Mak PI, Ham D (2020). Portable NMR with Parallelism. Anal Chem.

[bib1.bib43] Liong M, Hoang AN, Chung J, Gural N, Ford CB, Min C, Shah RR, Ahmad R, Fernandez-Suarez M, Fortune SM, Toner M, Lee H, Weissleder R (2013). Magnetic barcode assay for genetic detection of pathogens. Nat Commun.

[bib1.bib44] Liu Y, Sun N, Lee H, Weissleder R, Ham D (2008). CMOS mini nuclear magnetic resonance system and its application for biomolecular sensing.

[bib1.bib45] Minard KR, Wind RA (2001). Solenoidal microcoil design – Part II: Optimizing winding parameters for maximum signal-to-noise performance. Concept Magnetic Res.

[bib1.bib46] Morris GA (1988). Compensation of Instrumental Imperfections by Deconvolution Using an Internal Reference Signal. J Magn Reson.

[bib1.bib47] Morris GA, Barjat H, Horne TJ (1997). Reference deconvolution methods. Prog Nucl Mag Res Sp.

[bib1.bib48] Peng WK, Kong TF, Ng CS, Chen L, Huang YX, Bhagat AAS, Nguyen NT, Preiser PR, Han J (2014). Micromagnetic resonance relaxometry for rapid label-free malaria diagnosis. Nat Med.

[bib1.bib49] Rahman A-U, Choudhary MI, Wahab A-T (2016). Solving problems with NMR spectroscopy.

[bib1.bib50] Rudszuck T, Nirschl H, Guthausen G (2021). Perspectives in process analytics using low field NMR. J Magn Reson.

[bib1.bib51] Singh K, Blumich B (2018). Compact low-field NMR spectroscopy and chemometrics: A tool box for quality control of raw rubber. Polymer.

[bib1.bib52] Solmaz NS, Grisi M, Matheoud AV, Gualco G, Boero G (2020). Single-Chip Dynamic Nuclear Polarization Microsystem. Anal Chem.

[bib1.bib53] Stapf SHS-I (2010). NMR imaging in chemical engineering.

[bib1.bib54] Sun N, Liu Y, Lee H, Weissleder R, Ham D (2009). CMOS RF biosensor utilizing nuclear magnetic resonance. IEEE J Solid-St Circ.

[bib1.bib55] Sun N, Yoon T-J, Lee H, Andress W, Weissleder R, Ham D (2011). Palm NMR and 1-chip NMR. IEEE J Solid-St Circ.

[bib1.bib56] Takahashi M, Ebisawa Y, Tennmei K, Yanagisawa Y, Hosono M, Takasugi K, Hase T, Miyazaki T, Fujito T, Nakagome H, Kiyoshi T, Yamazaki T, Maeda H (2012). Towards a beyond 1 GHz solid-state nuclear magnetic resonance: External lock operation in an external current mode for a 500 MHz nuclear magnetic resonance. Rev Sci Instrum.

[bib1.bib57] Wu NA, Peck TL, Webb AG, Magin RL, Sweedler JV (1994). H-1-NMR Spectroscopy on the Nanoliter Scale for Static and Online Measurements. Anal Chem.

[bib1.bib58] Wu ZX, Lu RS, Jiang XW, Wang JN, Chen Y, Feng P, Xie ZH, Ni ZH, Yi H, Xiao D (2021). An NMR Relaxation Method of Characterizing Hydrogen-Bearing Crystalline Solid Phases in Hydrated Cement Paste. IEEE T Instrum Meas.

[bib1.bib59] Yang Q, Wang JN, Hu Z, Ni ZH, Lu RS, Yi H (2021). A low-cost, miniature Halbach magnet designed for portable time domain NMR. Int J Appl Electromagn Mech.

[bib1.bib60] Yu P, Xu YJ, Wu ZY, Chang Y, Chen QY, Yang XD (2018). A low-cost home-built NMR using Halbach magnet. J Magn Reson.

